# Effects of Adding Small Combat Games to Regular Taekwondo Training on Physiological and Performance Outcomes in Male Young Athletes

**DOI:** 10.3389/fphys.2021.646666

**Published:** 2021-04-01

**Authors:** Ibrahim Ouergui, Emerson Franchini, Hamdi Messaoudi, Hamdi Chtourou, Anissa Bouassida, Ezdine Bouhlel, Luca Paolo Ardigò

**Affiliations:** ^1^High Institute of Sport and Physical Education of Kef, University of Jendouba, Jendouba, Tunisia; ^2^Martial Arts and Combat Sports Research Group, School of Physical Education and Sport, University of São Paulo, São Paulo, Brazil; ^3^Institut Supérieur du Sport et de l'Education Physique de Sfax, Université de Sfax, Sfax, Tunisia; ^4^Activité Physique, Sport et Santé, UR18JS01, Observatoire National du Sport, Tunis, Tunisia; ^5^Laboratory of Cardio-Circulatory, Respiratory, Metabolic and Hormonal Adaptations to Muscular Exercise, Faculty of Medicine Ibn El Jazzar, University of Sousse, Sousse, Tunisia; ^6^Department of Neurosciences, Biomedicine and Movement Sciences, School of Exercise and Sport Science, University of Verona, Verona, Italy

**Keywords:** physiological responses, training, aerobic performance, agility, martial arts

## Abstract

This study investigated the effect of area sizes (4 × 4, 6 × 6, and 8 × 8 m) and effort-pause ratios (free combat vs. 1:2) variation on the physiological and perceptive responses during taekwondo combats (Study 1). In a second study, the effects on physical performance of 8 weeks of small combat-based training added to regular taekwondo training were investigated (Study 2). In random order, 32 male taekwondo athletes performed six (i.e., two effort-to-pause ratios × three area sizes conditions) different 2-min taekwondo combats (Study 1). Thereafter (Study 2), they were randomly assigned to three experimental groups (4 × 4, 6 × 6, and 8 × 8 m) and an active control group (CG). Regarding Study 1, blood lactate concentration [La] before and after each combat, mean heart rate (HRmean) during each combat, and rating of perceived exertion (CR-10) immediately after each combat were assessed. Regarding Study 2, progressive specific taekwondo (PSTT) to estimate maximum oxygen consumption (VO_2max_), taekwondo-specific agility, and countermovement jump (CMJ) tests were administered before and after 8 weeks of training. Study 1 results showed that 4 × 4 m elicited lower HRmean values compared with 6 × 6 m (*d* = −0.42 [small], *p* = 0.030) and free combat induced higher values compared with the 1:2 ratio (*d* = 1.71 [large], *p* < 0.001). For [La]post, 4 × 4 m area size induced higher values than 6 × 6 m (*d* = 0.99 [moderate], *p* < 0.001) and 8 × 8 m (*d* = 0.89 [moderate], *p* < 0.001) and free combat induced higher values than 1:2 ratio (*d* = 0.69 [moderate], *p* < 0.001). Higher CR-10 scores were registered after free combat compared with 1:2 ratio (*d* = 0.44 [small], *p* = 0.007). For Study 2, VO_2max_ increased after training [*F*_(1, 56)_ =30.532, *p* < 0.001; *post-hoc*: *d* = 1.27 [large], *p* < 0.001] with higher values for 4 × 4 m compared with CG (*d* = 1.15 [moderate], *p* = 0.009). Agility performance improved after training [*F*_(1, 56)_ = 4.419, *p* = 0.04; *post-hoc*: *d* = −0.46 [small], *p* = 0.04] and 4 × 4 m induced lower values in comparison with 6 × 6 m (*d* = −1.56 [*large*], *p* = 0.001) and CG (*d* = −0.77 [moderate], *p* = 0.049). No training type influenced CMJ performance. Smaller area size elicited contrasting results in terms of metabolic demand compared with larger sizes (i.e., lower HRmean but higher [La] and CR-10), whereas free combat induced variables' consistently higher values compared with imposed 1:2 ratio (Study 1). Taekwondo training is effective to improve VO_2max_ and agility (Study 2), but small combat training modality should be investigated further.

## Introduction

To improve taekwondo athletes' fitness level, coaches regularly use a variety of conventional activities including basic techniques, technical combinations, poomsae (forms), breaking techniques, self-defense techniques, step sparring, sparring drills, and free sparring that can be performed including additional materials such as elastics and pads (Bridge et al., 2007). These different exercise modalities were reported to stress the cardiovascular system at different intensities (Bridge et al., 2007). Specifically, the sparring drills and free sparring practice was reported to elicit greater demands on the cardiovascular system compared with other activities (e.g., elastics and technical combinations) making them particularly suitable for developing and maintaining the necessary cardiovascular fitness for successful performance (Bridge et al., 2007). Bridge et al. (2007) suggested that exercises based on combat drills and free combat are the most suitable activities for developing cardiovascular fitness specifically for taekwondo competition when compared with the elastics' use (basic techniques performed with elastic bands for extra resistance) and technical combinations (basic hitting techniques performed in various combinations). Moreover, when organizing sparring sessions, coaches can manipulate the combat area and/or the effort-to-pause ratio in an attempt to increase the athletes' number of attacks (Ouergui et al., 2019).

Taking into consideration these aspects, previous studies have investigated the physiological responses and perceived exertion in combat-based exercises in taekwondo (Bridge et al., 2009), kickboxing (Ouergui et al., 2017), and judo (Franchini et al., 2019). These studies investigated whether varied area size, number of within-combat sparring partners, or effort-to-pause ratio induce different physiological strains. Intensities achieved during different combinations were in the range recommended to develop cardiovascular fitness (Ouergui et al., 2017, 2019, 2021). However, there is no evidence that integrating these training modalities within traditional training programs can be an appropriate strategy to enhance the athletes' physical performance. Specifically with taekwondo, Ouergui et al. (2021) investigated the effects of varying area sizes (i.e., 4 × 4, 6 × 6, and 8 × 8 m) and within-round sparring partners and showed higher mean heart rate (HRmean) and percentage of maximum heart rate (%HRmax) values with 1 vs. 1 compared with 1 vs. 2 condition; but the effort-to-pause ratio was not manipulated in this study. For taekwondo youth athletes, Tornello et al. (2013) reported that fighting (i.e., attack or defense)-to-non-fighting (i.e., preparatory movements) combats ratio was 1:2. Therefore, effort-to-pause ratio should be addressed in such an exercise.

To the current authors' knowledge, there are no studies that investigated physiological and perceptive responses during small combat in youth taekwondo athletes by varying both area size and effort-to-pause ratio. Small combat is a special combat type with specific rules such as modified time, effort-to-pause ratios, area sizes, and/or the number of within-round sparring partners (Ouergui et al., 2017, 2021). Moreover, no studies that have examined the effects of training program using training modalities integrating small combat sessions into a traditional program on the taekwondo athletes' physical fitness (i.e., maximum aerobic power, agility, and jumping ability). Thus, this study aimed at examining (1) the physiological responses (heart rate [HR], blood lactate concentration [La]) and rating of perceived exertion (CR-10) during small combats in taekwondo using different area sizes (4 × 4, 6 × 6, 8 × 8 m) and effort-to-pause ratios (free combat, imposed 1:2 ratio), and (2) the effects of 8 weeks of training program enriched by small combat sessions on the athletes' physical performance. It was hypothesized that (1) the physiological and perceptive responses would be higher when using small area size (i.e., 4 × 4 m) and/or free combat and (2) adding small combat games using free ratio with small area size to taekwondo regular training regime would induce higher aerobic fitness and agility improvements (Bridge et al., 2007).

## Materials and Methods

### Participants

Thirty-two male taekwondo athletes (mean ± SD: age: 15 ± 1 yrs; height: 1.71 ± 0.64 m; body mass: 55.7 ± 6.8 kg; and taekwondo experience: 8 ± 2 yrs) competing at regional and national levels were recruited. Athletes trained regularly for an average of 8years with 3 training sessions of 2 h per week. Participants did not present any medical conditions, acute, or chronic injuries over the 6 months before—according to self-declarations—and during the whole experimentation period.

The study was conducted according to the Declaration of Helsinki for human experimentation (World Medical Association, 2013) and the protocol was fully approved by the University of Sfax research ethics committee (CCP: 19/024) before the beginning of the study. All athletes' parents and their coach gave written informed consent after a detailed explanation about the aims, benefits, and potential risks involved in the investigation.

All testing and training sessions were conducted with the presence of the athletes' coach to give specific feedbacks during sparring sessions to ensure that athletes perform with their best abilities.

### Procedures

#### Study 1

To examine the physiological and perceptive responses during taekwondo combats changing the temporal structure and area sizes, athletes were submitted randomly to 6 experimental conditions (i.e., six 2-min test rounds) organized in separated days and with a minimum interval of 48 h between each other. Specifically, two effort-to-pause ratios (free combat and imposed 1:2 ratio combat) combined with 3 area sizes (4 × 4, 6 × 6, and 8 × 8 m size) were administered to assess six (i.e., free combat in 4 × 4 m, free combat in 6 × 6 m, free combat in 8 × 8 m, imposed 1:2 ratio in 4 × 4 m, imposed 1:2 ratio in 6 × 6 m, and imposed 1:2 ratio in 8 × 8 m) different conditions. During imposed 1:2 ratio combat, the fighting, and non-fighting periods were manipulated by the coach. Briefly, during each round, after each fighting period (~6 s), athletes were stopped by the operator for a double time of the previous fighting period duration (~12 s). During free combat, athletes were not constrained to any prescription. This strategy was adopted to create situations that could modify the intensity of combats. Area sizes were chosen in order to simulate combat distances. Moreover, effort-to-pause ratios were used according to those reported in literature for this specific age group (Tornello et al., 2013). One week before the beginning of the investigation, athletes were familiarized with the tests and the sparring sessions and all experimental sessions were conducted at the same time of day to avoid any diurnal variation of the performance (Chtourou and Souissi, 2012). Hereafter, athletes accomplished the 20 multistage shuttle run test (Léger et al., 1988) to determine HRmax, which was the highest HR value achieved during this test. Experimental sessions were directed by two investigators to ensure safety and maximum effort during combats. During all conditions, HR (continuously), [La] (before and after each condition), and CR-10 (after each condition) were recorded/measured. Before each experimental session, athletes performed 15 min of a standardized warm-up session consisting of jogging and dynamic stretching, after which 3 min of passive rest and baseline measures were taken.

#### Physiological and Perceptive Measurements

Blood samples were taken 10 min before and immediately after each condition from the fingertip and [La] was measured using the Lactate Pro2 Analyzer (Arkray, Tokyo, Japan). Blood lactate concentration at pre-and post-conditions was used for the analyses. Pre-condition blood lactate concentrations were checked only to assess the same inter-group pre-condition values. Heart rate was measured every 5 s throughout the taekwondo combat sessions (Polar Team2 Pro System, Polar Electro OY, Kempele, Finland) and HRmean was used for the analysis. After being familiarized with the scale (i.e., being thoroughly taught and assessed regarding it), athletes were asked to report their CR-10 scores (Foster et al., 2001) after each combat session.

#### Study 2

Study 2 aimed at investigating the effect of an added training program according to small combat games on maximum oxygen consumption (VO_2max_) and taekwondo related performance variables such as agility and jumping performances. Athletes were randomly assigned either to a control group (CG) or one of three experimental groups based on area size variation in three manners: 4 × 4, 6 × 6, and 8 × 8 m. Athletes in experimental groups were from similar weight categories and completed additional training according to sparring sessions in their assigned area size twice a week during an 8-week training period in addition to their usual technical-tactical taekwondo training; whereas the CG followed just its regular taekwondo training. Athletes were asked to avoid any strenuous exercises in the day preceding all test sessions. All athletes were familiarized with the experimental procedures of pre- and post-tests in a specific session 1 week before the beginning of the pre-tests. Furthermore, athletes performed the following tests: the progressive specific taekwondo test (PSTT; Sant' Ana et al., 2019), the taekwondo-specific agility test (TSAT; Chaabene et al., 2018), and countermovement jump test (CMJ; Marković et al., 2008).

#### Training Intervention

The training intervention for the experimental groups consisted of adding twice a week 2 blocks of sparring sessions separated by 4 min of rest and contested in different area sizes (i.e., 4 × 4, 6 × 6, and 8 × 8 m). Each block consisted of 3 rounds of 2 min with 1 min of rest in between. The CG underwent the same habitual taekwondo training sessions as the experimental groups without additional training over the study duration.

#### Progressive Specific Taekwondo Test

The PSTT is a progressive maximal graded test until voluntary exhaustion to assess aerobic power and capacity in athletes during a taekwondo-specific effort. The PSTT was performed on a 2 × 2 m pitch using a punching bag of 1.0 × 0.9 m. In the first stage, participants began with the right leg and performed 6 kicks for 100 s with alternating legs. In the subsequent stages, the number of kicks increased by 4 kicks per stage while the duration of the stages decreased proportionally. During the test, athletes remained in step position (hopping in fighting position). The kicking frequency was dictated by acoustic signals with fixed intervals between each kick over each stage (Sant' Ana et al., 2019). During the PSTT, HR was recorded continuously at 0.5 s intervals using HR monitors (Polar Team2 Pro System, Polar Electro, Kempele, Finland) and HRmean was calculated. Maximum oxygen consumption was indirectly calculated based on the prediction equation determined by Rocha et al. (2016). Progressive specific taekwondo test was administered twice, once immediately after the familiarization and another time on occasion of pre-test, for the intraclass correlation coefficient calculation purpose. Intraclass correlation coefficient for the test-retest trial of the present study was 0.80.

#### Taekwondo-Specific Agility Test

The athlete began the test in his/her fighting position behind the start line. At his discretion, the athlete advanced to the center mark as fast as possible. Following his own choice, he turned toward partner 1 by a shift and performed a roundhouse kick with his lead leg. Then he turned to the other side and shifted to partner 2 and performed another roundhouse kick with the other lead leg. Thereafter, he returned to the center, moved to partner 3 in the guard position, and performed a double roundhouse kick. Finally, the athlete ran backward to the start/finish line (Chaabene et al., 2018). The performance time was measured with two sets of single beam timing lights (Brower Timing Systems, Salt Lake City, UT, USA). Three trials were performed by each athlete and the best performance was used for the analysis and two min of passive rest were allowed between trials. Taekwondo-specific agility test was administered twice, once immediately after familiarization and another time on occasion of the pre-test, for intraclass correlation coefficient calculation purpose. Intraclass correlation coefficient for the test-retest trial of the present study was 0.82.

#### Countermovement Jump Test

Athletes started from an upright standing position, completed a fast-downward movement by flexing the knees and hips immediately followed by a rapid extension of both legs. During the extension of the legs, a vertical arm-swing of both arms was performed for better performance in the CMJ (i.e., it was an arm-aided CMJ; Marković et al., 2008). The performance was recorded as the height of the jump using a photocell device (Optojump, Microgate, Bolzano, Italy). Three trials were performed and the best performance was used for the analysis. Forty-five seconds of passive recovery was allowed between trials. CMJ jump test was administered twice, once immediately after the familiarization and another time on occasion of the pre-test, for intraclass correlation coefficient calculation purpose. Intraclass correlation coefficient for the test-retest trial of the present study was 0.93.

### Statistical Analyses

Data are presented as mean and standard deviation. Statistical analysis was performed using SPSS 20.0 statistical software (IBM, Armonk, NY, USA). The normality of data sets was checked and confirmed using the Kolmogorov-Smirnov test. Sphericity was tested and confirmed using the Mauchly test. For the Study 1, data were analyzed using a two-way analysis of variance (ANOVA, area size [4 × 4, 6 × 6, and 8 × 8 m] × ratio (free combat and imposed 1:2 ratio]) to compare HR values, [La] and CR-10. For the Study 2, performance at pre-training was compared between groups using a one-way ANOVA and results showed no significant baseline difference between groups. Data were thus analyzed using a two-way ANOVA (training groups [4 × 4, 6 × 6, and 8 × 8 m] × time-point [before and after training]) to compare the tests' outcomes. When a significant difference was found, Bonferroni was used as *post-hoc* test. The magnitude of differences between variables was interpreted as standardized effect size (Cohen's *d*) and classified according to Hopkins^1^ *d* ≤ 0.2 (trivial); 0.2 < *d* ≤ 0.6 (small); 0.6 < *d* ≤ 1.2 (moderate); 1.2 < *d* ≤ 2.0 (large); 2.0 < *d* ≤ 4.0 (very large) and >4.0 (extremely large). Moreover, upper and lower 95% confidence intervals of the difference (95% CI_d_s) were calculated for the corresponding variation. The statistical significance level was set at *p* ≤ 0.05.

## Results

### Study 1

The mean value for HRmax recorded during the multistage 20-m shuttle run test was 199 ± 9 beats·min^−1^. Table 1 presents the physiological and CR-10 values recorded during different experimental conditions. For HRmean, there was an area size effect [*F*_(2, 138)_ = 3.667, *p* = 0.028] with the 4 × 4 m eliciting lower values compared with 6 × 6 m (95%CI_*d*_ = −10, −1, *d* = −0.42 [small], *p* = 0.030). Likewise, ratio-structure effect was found [*F*_(1, 138)_ = 109.247, *p* < 0.001] with the free combat inducing higher values compared with the imposed 1:2 ratio (95%CI_*d*_ = 15,22, *d* = 1.71 [large], *p* < 0.001) and no interaction effect [*F*_(2, 138)_ = 0.631, *p* = 0.534] was reported.

**Table 1:** Physiological responses and rating of perceived exertion values recorded during different experimental conditions (values are mean ± SD, *n* = 32).

	**[La]pre (mmol/l)**	**[La]post (mmol/l)**	**HRmean (beats·min^**−1**^)**	**HR (%HRmax)**	**CR-10 (a.u.)**
**Free combat**					
4 × 4 m	2.3 ± 0.6	9.4 ± 1.2^b^^,^ ^c^^,^ ^d^	157 ± 10^a^^,^ ^b^	79 ± 5	6 ± 1^e^
6 × 6 m	3.0 ± 1.0	8.1 ± 1.2^d^	161 ± 9^b^	81 ± 5	6 ± 2 ^e^
8 × 8 m	2.9 ± 0.8	8.0 ± 1.4^d^	160 ± 10^b^	80 ± 6	5 ± 1 ^e^
**Imposed 1:2** ***ratio***					
4 × 4 m	2.2 ± 0.7	8.3 ± 1.1^b^^,^ ^c^	160 ± 12^a^	70 ± 7	5 ± 1^e^^,^ ^f^
6 × 6 m	2.4 ± 0.6	6.9 ± 1.5	160 ± 16	73 ± 3	4 ± 1
8 × 8 m	2.3 ± 0.8	7.5 ± 1.1	161 ± 16	70 ± 6	5 ± 1

a *area size main effect: HR mean was higher in 4 × 4 m compared with 6 × 6 m (p = 0.03)*,

b *ratio-structure main effect: HR mean was higher in free combat compared with imposed ratio (p < 0.001)*,

c *area size main effect: [La]post was higher in 4 × 4 m compared with 6 × 6 and 8 × 8 m (p < 0.001)*,

d *ratio-structure main effect: [La]post was higher in free combat compared with imposed ratio (p < 0.001)*,

e *ratio-structure main effect: CR-10 was higher in the free combat compared with imposed ratio (p = 0.007)*,

f *interaction effect between area size and ratio-structure: CR-10 was higher in 4 × 4 m for 1:2 ratio compared with 6 × 6 m for the same ratio (p = 0.012). a.u. arbitrary unit, [La]: blood lactate concentration, HRmean, mean heart rate, %HRmax, percentage of maximum heart rate*.

For [La]pre, there was no effect of area size [*F*_(2, 138)_ = 0.189, *p* = 0.828], combat ratio [*F*_(1, 138)_ = 0.012, *p* = 0.913], or interaction effect [*F*_(2, 138)_ = 0.311, *p* = 0.734] confirming the similar inter-groups pre-condition values. For [La]post, an area size main effect was found [*F*_(2, 138)_ = 16.068, *p* < 0.001] with the 4 × 4 m area size inducing higher values than 6 × 6 m (95%CI_*d*_ = 0.7,1.9, *d* = 0.99 [moderate], *p* < 0.001) and 8 × 8 m (95%CI_*d*_ = 0.5,1.7, *d* = 0.89 [moderate], *p* < 0.001). Likewise, a main effect of ratio-structure [*F*_(1, 138)_ = 21.061, *p* < 0.001] was found, with the free combat inducing higher values than imposed 1:2 ratio (95%CI_*d*_ = 0.5,1.3, *d* = 0.69 [moderate], *p* < 0.001). However, no interaction effect was revealed (*F*_(2, 138)_ = 1.144, *p* = 0.321].

For CR-10, no area size effect was found [*F*_(2, 138)_ = 2.731, *p* = 0.069]. However, a ratio-structure main effect was found [*F*_(1, 138)_ = 7.387, *p* = 0.007] with free combat inducing higher values compared with the imposed 1:2 ratio (95%CI_*d*_ = 0.2,1, *d* = 0.44 [small], *p* = 0.007). An interaction effect was found [*F*_(2, 138)_ = 3.769, *p* = 0.025] with the 4 × 4 m area size for 1:2 ratio inducing higher values compared with the 6 × 6 m area size for the same ratio (95%CI_*d*_ = 0.2,2, *d* = 0.86 [moderate], *p* = 0.012).

### Study 2

Table 2 presents the performance recorded during PSTT, TSAT, and CMJ before and after the training period. For pre-training values, no statistically significant differences were found for VO_2max_ [*F*_(2, 63)_ = 1.58, *p* = 0.2], TSAT [*F*_(2, 63)_ = 0.52, *p* = 0.67], and CMJ [*F*_(2, 63)_ = 0.11, *p* = 0.95].

**Table 2:** Performance variables before and after the training period (values are mean ± SD) for the 4 × 4 m group (4 × 4 m and *n* = 8), the 6 × 6 m group (6 × 6 m and *n* = 8), the 8 × 8 m group (8 × 8 m and *n* = 8) and the control group (CG and *n* = 8).

	**PSTT/VO**_****2max****_**(ml/kg/min)**		**TSAT/Agility (s)**		**CMJ test/Jump height (cm)**	
	**Pre-training**	**Post-training**	**Pre-training**	**Post-training**	**Pre-training**	**Post-training**
4 × 4m	47.0 ± 1.8^b^	53.7 ± 4.6^a^^,^ ^b^	6.7 ± 0.1^d^^,^ ^e^	6.3 ± 0.4^c^^,^ ^d^^,^ ^e^	26.1 ± 3.6	27.4 ± 3.4
6 × 6m	46.1 ± 3.2	51.3 ± 3.6^a^	7.0 ± 0.4	7.1 ± 0.4	26.0 ± 2.3	25.9 ± 2
8 × 8m	47.0 ± 3.2	49.2 ± 3.5^a^	6.8 ± 0.2	6.8 ± 0.3	25.9 ± 2.5	26.3 ± 1.4
**CG**	45.4 ± 1.5	47.6 ± 1.4^a^	7.2 ± 0.5	6.6 ± 0.6^c^	26.9 ± 4.7	26.5 ± 5.3

a *main time-point effect: VO_2max_ post differed from pre-training (p < 0.001)*,

b *main group effect: 4 × 4 m differed from CG (p = 0.009)*,

c *main time-point effect: agility post differed from pre-training (p = 0.04)*,

d *main group effect: 4 × 4 m differed from 6 × 6 m (p = 0.001)*,

e *main group effect: 4 × 4 m differed from CG (p = 0.049). PSTT, progressive specific taekwondo test; VO_2max_, estimated maximum oxygen consumption; TSAT, taekwondo specific agility test; CMJ, countermovement jump*.

Regarding estimated VO_2max_ in the PSTT, it was found a time-point effect [*F*_(1, 56)_ = 30.532, *p* < 0.001) with increased values after in comparison with before training (95%CI_*d*_ = 2.4,5.1, *d* = 1.27 [large], *p* < 0.001). A training group effect was found [*F*_(3, 56)_ = 3.871, *p* = 0.014], with 4 × 4 m eliciting higher values compared with CG (95%CI_*d*_ = 0.6,5.9, *d* = 1.15 [moderate], *p* = 0.009). However, no interaction effect was found [*F*_(3, 56)_ = 1.747, *p* = 0.168].

Regarding TSAT performance, it was found a time-point effect [*F*_(1, 56)_ = 4.419, *p* = 0.04] with values decreasing after compared with before training (95%CI_*d*_ = −0.4, −0.01, *d* = −0.46 [small], *p* = 0.04). It was found a training group effect [*F*_(3, 56)_ = 5.899, *p* = 0.001] with 4 × 4 m inducing lower values in comparison with 6 × 6 m (95%CI_*d*_ = −0.9, −0.2, *d* = −1.56 [large], *p* = 0.001) and CG (95%CI_*d*_ = −0.8, −0.01, *d* = −0.77 [moderate], *p* = 0.049). However, no interaction effect was found [*F*_(3, 56)_ = 2.602, *p* = 0.061].

Regarding CMJ performance, no time-point [*F*_(1, 56)_ = 0.146, *p* = 0.704], training group [*F*_(3, 56)_ = 0.233, *p* = 0.873], or interaction effects [*F*_(3, 56)_ = 0.211, *p* = 0.889] were found.

## Discussion

### Study 1

The results of the present study showed that the 4 × 4 m area size elicited lower HRmean values compared with the 6 × 6 m and that the free combat induced higher values compared with the imposed 1:2 ratio. Regarding post-combat sessions [La], the 4 × 4 m area size induced higher values compared with 6 × 6 and 8 × 8 m, and the free combat induced higher values compared with the imposed 1:2 ratio. As for the CR-10 values, the free combat induced higher values compared with the imposed 1:2 ratio and, for 1:2 ratio, 4 × 4 m area size induced higher values compared with 6 × 6 m.

Considering HR values, the intensities recorded during different conditions ranged from 70 to 81% HRmax, situated in the range of intensity suitable to induce cardiovascular and respiratory improvements (Garber et al., 2011). Similarly, Bridge et al. (2007) showed that varied taekwondo activities resulted in HR values ranging from 64.7 to 81.4% HRmax and exercises based on sparring sessions and free combat were the activities that elicited the highest HR values (80.8 and 81.4% HRmax, respectively). Considering the area size effect, HRmean values were lower in the 4 × 4 m area size compared with the 6 × 6 m. Contrarily, Ouergui et al. (2021), in taekwondo athletes, showed that HRmean and %HRmax did not vary according to area sizes. This difference may be due to the difference in terms of age categories of athletes who participated in the two studies. In another study, Ouergui et al. (2017) showed that HRmean values during kickboxing small combat games did not vary according to area sizes, whereas 4 × 4 m elicited a higher percentage of peak HR compared to small (2 × 2 m) and large (6 × 6 m) area sizes. Regarding the same issue, a recent study on female judo athletes showed that HRmean values did not vary according to area size variation (Ouergui et al., 2019). Moreover, in terms of the time structure effect, free combat induced higher HR values compared with the imposed 1:2 ratio. Despite the temporal structure of free combats was not investigated in the present study, this result may be the cause of a shorter effort-to-pause ratio and a high amount of activity during free combat vs. imposed 1:2 ratio, which induced higher cardiovascular demands in the free combat compared with the imposed 1:2 ratio. Contrarily to our result, Ouergui et al. (2019) showed that HRmean values did not vary according to the time structure variation.

Regarding [La], the present study showed that values ranged approximatively from 7 to 10 mmol·l^−1^ and resulted in lower concentrations than those recorded during small combats in junior taekwondo athletes (11.44 ± 2.78 vs. 14.55 ± 3.84 mmol·l^−1^) showing that the age categories may be an influencing factor for [La] production during small combat games as reported in taekwondo competitions (Herrera Valenzuela et al., 2014). However, [La] values were similar to those recorded in other studies in taekwondo competitions (Bouhlel et al., 2006; Butios and Tasika, 2007; Campos et al., 2012). The results of the present study showed that [La]post combats was higher in the 4 × 4 m compared with the other area sizes and during the free combat compared with the imposed 1:2 ratio showing that the absolute anaerobic glycolysis solicitation was higher in those conditions. This disagrees with the results reported by Ouergui et al. (2021) in taekwondo and Ouergui et al. (2017) in kickboxing athletes. Indeed, these studies reported that area size was not a factor contributing to the variation in [La]. However, Ouergui et al. (2019) showed that the 4 × 4 m area induced higher [La] values compared with the other spaces during women's judo matches. This can be explained by the fact that this area was the most proper to induce more contacts and actions during combats and thus resulting in [La] increases (Ouergui et al., 2019). Likewise, Ouergui et al. (2019) showed that [La] did not vary in terms of effort-to-pause ratio variation.

In respect to CR-10, values ranged from 4 ± 1 to 6 ± 1 a.u. showing that the present study's experimental conditions were perceived as from “somewhat difficult” to “difficult” activities. A recent study on small combats in taekwondo athletes showed that CR-10 scores ranged from 5 ± 1 to 7 ± 1 a.u. (Ouergui et al., 2021). The present study showed that CR-10 did not vary according to area sizes. Similar results from recent studies in taekwondo (Ouergui et al., 2021) and kickboxing (Ouergui et al., 2017) athletes also showed that perceived exertion was not altered according to different area sizes. Differently, Ouergui et al. (2019) reported that the rating of perceived exertion scores were higher in 4 × 4 m comparably with other area sizes in female judo combats. This difference may be due to the different nature of these sports (striking vs. grappling) and to the different subjects in these studies (males vs. females). In fact, while 4 × 4 m can be a suitable area to increase time in grip dispute, as the athletes have a lower space between them in grappling sports (Ouergui et al., 2019), this size may be unsuitable to deliver a high amount of kicks and punches and reach the target several times. Regarding this, it was reported that adults—compared with young athletes—in taekwondo competition used more tactics based on avoiding unnecessary displacement (Herrera Valenzuela et al., 2014). Further technical-tactical analyses during these small combats are strongly needed to provide more explanations. The present study showed that free combat induced higher CR-10 values compared with the imposed 1:2 ratio. Differently, Ouergui et al. (2019) showed that perceived exertion scores in female judo combats were not altered by the variation of the effort-to-pause ratio.

### Study 2

The present study showed that VO_2max_ estimated from PSTT increased after the training period and that 4 × 4 m elicited higher values compared with CG. Moreover, agility performance improved after the training period and 4 × 4 m induced lower values in comparison to 6 × 6 m and CG. Aerobic fitness has been reported to be an important factor that positively affects the taekwondo athletes' performance by enhancing their recovery process, which is paramount in performing successive high- intensity actions (Monks et al., 2017). In this regard, the present study showed that VO_2max_ values increased after the training program. These results are not in agreement with those reported by other studies (Melhim, 2001; Kim et al., 2011), which showed that 12 weeks (2 sessions per week; Kim et al., 2011) or 8 weeks (3 sessions per week; Melhim, 2001) of habitual taekwondo training were not able to improve VO_2max_, suggesting that only taekwondo-based training was not a sufficient *stimulus* to improve cardiorespiratory fitness.

Regarding agility, the present study showed that performance improved after the training period with the 4 × 4 m group values being different from the 6 × 6 m and the CG. Differently, it was reported that 12 weeks of regular taekwondo training were not able to induce improvement in speed and agility performances (Kim et al., 2011). This difference may be explained by the difference in terms of subjects (untrained females vs. trained males) and tests used within the two studies (generic vs. specific agility tests). Furthermore, specific agility performance's improvement recorded during the present study may be mainly the result of the movements' repetition during combats which are similar to those characterizing the TSAT (i.e., lateral displacements combined with taekwondo kicks; Chaabene et al., 2018). Moreover, the 4 × 4 m training group induced improvement compared with 6 × 6 m and CG. These results should be interpreted with caution because studies in this area of investigation are lacking. Thus, the above-mentioned difference was only a main effect of group and cannot be attributed to any specific adaptation in response to the specific 4 × 4 m training mode. More studies are needed to verify which factors effectively affected this issue.

Concerning CMJ performance, jumping height did not improve after the training period. Contrarily to the present study's results, Kim et al. (2011) reported lower limbs power improvement using standing long jump following 12 weeks of a regular taekwondo training program in adolescent females. This difference may be attributed to the training duration [i.e., longer in the Kim's et al. (2011) study] as well as the different levels of subjects participating in the two studies (untrained vs. trained subjects). The lack of improvement shows that training programs based on small combats may be lacking the necessary overload and specific movements to develop muscle power and only technical aspects of jumping performance should be practiced when power improvement is pursued (Tolfrey and Smallcombe, 2017). Moreover, it can be concluded that muscle power cannot be improved using sport specifications that are not enough to induce improvements despite they are practiced for a longer time.

This study presents some limitations regarding the control of the nutritional intake, the sleep quantity and quality, and how much effort the athletes put into both the training and the testing periods. However, the influence of these aspects should have been similar across the groups investigated in the present study. In fact, technical-tactical aspects, as well as ratio-structure were not investigated in more detail to provide more explanations of the physiological responses during the different experimental conditions. Future studies could investigate the effects of age-specific ratio-structure manipulation on taekwondo performance, and on athletes of different competitive levels. Additional research could consider longer training programs and controlling samples for total training volume, as well.

## Conclusions

According to Study 1, it was found that the 4 × 4 m area size elicited lower HRmean values compared with the 6 × 6 m and that free combat induced higher values compared with the imposed 1:2 ratio. Regarding [La] post-combat, the 4 × 4 m area size induced higher values compared with 6 × 6 and 8 × 8 m area sizes, and the free combat induced higher values compared with the imposed 1:2 ratio. Regarding CR-10 values, the free combat induced higher values compared with the imposed 1:2 ratio, and for the 1:2 ratio, the 4 × 4 m area size induced higher values compared to 6 × 6 m. Furthermore, according to Study 2, VO_2max_ values collected with PSTT increased after the training period, and the 4 × 4 m group elicited higher values compared with CG. Moreover, the agility performance improved after the training period, and the 4 × 4 m group induced lower values in comparison with the 6 × 6 m group and the CG. No training type influenced CMJ performance. This study does not explain specific adaptations occurring in response to the investigated specific training mode (i.e., small combat). Yet, coaches can structure adequately their training programs, optimize taekwondo competitors' performance by programming sparring drills or adding free combats to regular taekwondo training regime during specific phases when combining physical, physiological, technical-tactical aspects is needed. Finally, further studies in this area are needed to provide more information about this training modality.

## Data Availability Statement

The raw data supporting the conclusions of this article will be made available by the authors, without undue reservation.

## Ethics Statement

The studies involving human participants were reviewed and approved by University of Jendouba, Human Research Ethics Committee. Written informed consent to participate in this study was provided by the participants' legal guardian/next of kin.

## Author Contributions

All authors listed have made a substantial, direct and intellectual contribution to the work, and approved it for publication.

## Conflict of Interest

The authors declare that the research was conducted in the absence of any commercial or financial relationships that could be construed as a potential conflict of interest.

## Footnotes

^1^A New View of Statistics: Effect Magnitudes, https://www.sportsci.org/resource/stats/effectmag.html.
